# The Diagnostics and Treatment of Recurrent Pregnancy Loss

**DOI:** 10.3390/jcm12144768

**Published:** 2023-07-19

**Authors:** Julia Tomkiewicz, Dorota Darmochwał-Kolarz

**Affiliations:** 1 Fryderyk Chopin University Hospital No 1, 35-055 Rzeszow, Poland; 2 Department of Obstetrics & Gynecology, Medical College, University of Rzeszow, 35-959 Rzeszow, Poland; ddarmochwal@ur.edu.pl

**Keywords:** recurrent pregnancy loss, RPL, pregnancy, live birth, RPL treatment, RPL diagnostic

## Abstract

Recurrent pregnancy loss is a common problem in the reproductive age population of women. It can be caused by many different conditions. This problem is addressed in international guidelines that take a slightly different approach to its diagnosis and treatment. The guidelines used in this review mainly use the guidelines of the Royal College of Obstetricians and Gynaecologists (RCOG), American Society of Reproductive Medicine (ASRM) and European Society of Human Reproduction and Embryology (ESHRE). This review shows how much the approach to miscarriages has changed and how much more needs to be explored and refined. The review also addresses the topic of unexplained pregnancy loss, which continues to be a challenge for clinicians.

## 1. Introduction

Recurrent pregnancy loss (RPL) is a common problem in the reproductive age population of women. It is a challenging clinical issue associated with psychological aspects. Miscarriages are very difficult for patients, their partners and clinicians. The management of the problem can be different; it depends on the cause of RPL. This issue was considered in guidelines published by the Royal College of Obstetricians and Gynaecologists (RCOG) in 2011 [1], American Society of Reproductive Medicine (ASRM) in 2012 [2] and European Society of Human Reproduction and Embryology (ESHRE) in 2017 [3,4]. All guidelines define several causes of recurrent pregnancy loss such as parental genetic disorders, uterine anatomical malformations, problems with the endocrine system, hemostatic dysfunctions and others. Many causes of miscarriages are being found, and it can be treated, but still, about 50% of RPL cases remain unexplained.

## 2. Materials and Methods

This review was based on available data collected from the PubMed database, Google Scholar database and many different databases. The research was conducted by looking through keywords such as: “recurrent pregnancy loss”, “RPL”, “pregnancy”, “live birth”, “RPL treatment” and “RPL diagnostic”. The materials were collected from November 2022 to May 2023.

This review compares three guidelines (RCOG, ASRM and ESHRE) for recurrent pregnancy loss. Their methodological similarities and differences are summarized to compare definitions and clinical recommendations.

## 3. Definition of Recurrent Pregnancy Loss

A definition of miscarriage presented by the Royal College of Obstetricians and Gynaecologists guidelines, which were published in 2011, is a spontaneous loss of pregnancy in the uterus before the fetus reaches viability. It means that miscarriage happens until the 24th week of gestation. RCOG, in their Green-top Guideline No. 17, says that RPL is a miscarriage of three or more pregnancies. Moreover, the updated guideline states that miscarriages do not have to be consecutive to be defined as recurrent pregnancy loss. It affects 1% of couples trying to have a child. It is estimated that 1–2% of pregnancies in the second trimester of pregnancy will miscarry before the 23rd week and the 6th day of pregnancy [1,5]. The proposed definition of RPL is based on earlier research from 1990 published in The Lancet, which summarizes the results of epidemiological studies. The Lancet suggests that data should be collected up to 28 weeks of gestation, but it should also be possible to assess up to 20–22 weeks or a 500 g fetal weight [6].

In 2012, a publication of the American Society of Reproductive Medicine gave a definition of RPL as a miscarriage of two or more failed clinical pregnancies. ASRM estimates that the loss of two pregnancies will happen to 5% of women, and the loss of three or more pregnancies happens to about 1% [2].

The ESHRE from 2017 defines RPL as two or more pregnancies that do not have to be consecutive [3,4].

Another aspect of miscarriage that should be considered is how we understand the definition of pregnancy. ASRM includes in its definition the importance of ultrasounds and the histological confirmation of pregnancy [2], whereas the RCOG understands pregnancy from conception, which means that it includes biochemical pregnancy [1]. In ESHRE’s opinion, pregnancy needs confirmation with the use of serum or urinary chorionic gonadothropin, and it does not include ectopic and molar pregnancies [3,4]. It is important to consider because the risk of losing biochemical pregnancies is higher than that of losing clinical pregnancies (22% vs. 0.3%) [7]. ESHRE, to provide the definition, used the results of a study by van den Boogaard et al. in 2013 on whether the pregnancy losses are consecutive or whether two versus three losses are not associated with the risk of antiphospholipid syndrome (APS) [8], research by van den Boogaard et al. in 2010 which states that there is no difference in the possibility of carrier status (of a structural chromosomal abnormality) between couples who had two or three consecutive pregnancy losses and those who had two or three non-consecutive losses [9] and the study by Egerup et al. in 2016, which showed that there is some proof that whether the pregnancy losses are consecutive or not has an impact on prognosis in unexplained RPL [10].

There are some other definitions of recurrent pregnancy loss. One of them was offered by the French National College of Obstetricians and Gynecologists (CNGOF) in 2016. The CNGOF defines RPL as a history of three consecutive (or more) early miscarriages before the 14th week of gestation [11]. Another definition was accepted by the consensus of German Society of Gynecology and Obstetrics (DGGG), the Austrian Society of Gynecology and Obstetrics (ÖGGG) and the Swiss Society of Gynecology and Obstetrics (SGGG). The DGGG, the ÖGGG and the SGGG adopt the definition presented by the WHO, which defines recurrent pregnancy loss as three consecutive miscarriages before 20 weeks of gestation [12].

RPL definitions used in individual guidelines are compared in Table 1 [1,2,4].

## 4. Epidemiology

According to differences in the definitions of recurrent pregnancy loss, it is difficult to calculate the frequency of the problem. Miscarriages are a common problem which happen in one in four pregnancies [13], but RPL frequency is hard to define. It is assumed that the frequency of RPL occurs in 1–3% [7]. Due to RCOG’s guidelines, the most important risk factors for RPL are the mother’s age and the number of previous miscarriages. An advancing mother’s age is associated with quality and a lower number of oocytes. The research has shown that women aged 12–19 have a 13% risk of miscarriage, those aged 20–24 have an 11% risk, those aged 25–29 have a 12% risk, those aged 30–34 have a 15% risk, those aged 35–39 have a 25% risk, those aged 40–44 have a 51% risk and those aged ≥45 have a 93% risk. Moreover, an advanced father’s age also gives a higher risk of a miscarriage. Another issue that is taken into consideration is past reproductive history. The risk of further miscarriages is higher after each miscarriage, and after the third miscarriage, it reaches 40%. Also, the prognosis gets worse with the increase in the mother’s age [1].

Determining the epidemiology of RPL is difficult because most studies focus on spontaneous miscarriage, not RPL.

**Scheme 1:**
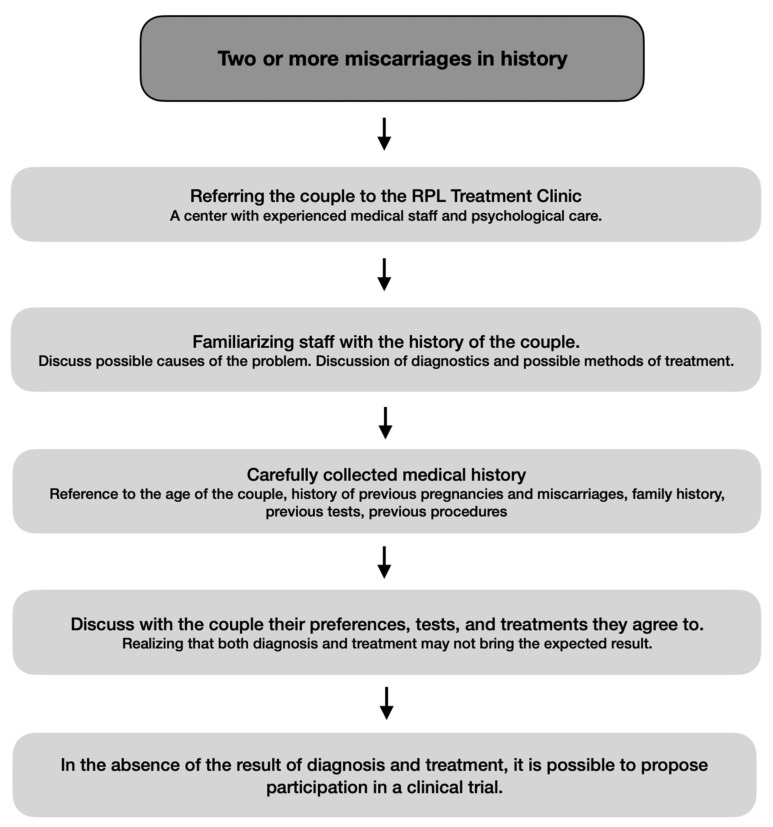
Organization of care for couples with RPL [4].

Couples affected by RPL should receive tailored help. The procedure for diagnosis and treatment is included in Scheme 1 [4].

## 5. Etiology

### 5.1. Chromosomal Factors

According to studies, we can divide chromosomal causes into parental chromosomal abnormalities and fetal chromosomal abnormalities [1]. About 50% of chromosomal abnormalities are a cause of pregnancy loss. It is said that there are other genetic abnormalities that are not currently possible to find. Usually, cytogenetic analysis of pregnancy tissue has to be conducted to identify a possible reason for pregnancy loss and to find out if there is a need to screen parents for genetic changes in their karyotype. It is important to identify any numeric chromosome errors. This issue is significant because these are causes of pregnancy loss that usually occur sporadically and do not have negative effects in further pregnancies, but if one of the parents has chromosomal aberrations, it is likely to occur in a future pregnancy [14]. The most common genetic abnormality in couples is balanced translocation, which can be found in 2–4% of couples with RPL. This cause can be detected by peripheral karyotyping in parents who are usually asymptomatic. During fertilization, a normal karyotype or a karyotype with a balanced or unbalanced translocation may be formed. Usually, pregnancies with unbalanced translocations end with a miscarriage, a stillbirth or a birth of an alive newborn with congenital defects. Despite the higher risk of genetic abnormalities in couples with an unbalanced translocation, the majority of them give birth to healthy children [1].

According to fetal genetic causes of miscarriages, the most common cause is embryonic aneuploidy, such as trisomy (60%), polyploidy (20%) and monosomy X (20%) [15]. The risk substantially increases with the mothers’ age. Most cases are de novo errors, so the risk of another aneuploidy in a further pregnancy is low. Moreover, the higher the number of pregnancy losses, the lower the possibility of a chromosomal abnormality cause [16]. In cases of seemingly euploid karyotypes, there may be genetic aberrations causing the loss of pregnancy that are not actually known or routinely checked. In these cases, the sequencing or analysis of microarrays should be used [14]. Studies conducted in 2020 showed no differences in the frequency of chromosomal aberrations found in the tissues of miscarriages between those with sporadic miscarriages and those with RPL [17]. In the future, the genetic analysis of embryonic pregnancy loss may give a chance to identify genes that play a key role in early human growth or whose lack of function leads to miscarriage [14].

### 5.2. Anatomical Factors

#### 5.2.1. Uterine Anomalies

Anatomical anomalies can be a reason for recurrent pregnancy loss. We can divide anatomical factors into congenital and acquired anomalies. The reported frequency of uterine anatomical anomalies in RPL populations is 1.8% and 37.6%. Differences in the percentage depend on the criteria and techniques that are used for diagnostics and also depend on which of the definitions is taken.

Congenital defects are a consequence of the abnormal progress of the Müllerian duct, which includes the septate, bicornuate, unicornuate, didelphic and arcuate uterus. They are reported in up to 10% of patients with recurrent pregnancy loss [18]. A uterus with a septum is the most common anomaly that causes miscarriage. Studies say that metroplasty increases the chances of pregnancy; therefore, it is recommended to delete the septum in patients with RPL [19]. The prevalence of uterine malformations seems to be higher in patients who miscarry in the second trimester compared with that of patients who suffer from a first-trimester pregnancy loss. There are reports that female patients with an arcuate uterus are more likely to lose the pregnancy in the second trimester of pregnancy, just like how women with a septate uterus are more likely to have a pregnancy loss in the second trimester [1].

Women who suffer from RPL can also have acquired uterine anomalies. These can be intrauterine adhesions, endometrial polyps and myomas. Intrauterine adhesions occur in places where the endometrial basal layer was destroyed; it can happen during uterine surgery, curettage, infection and complications in childbirth. Myomas are being classified by their location in the uterus (submucosal, intramural, subserosal), and it may cause miscarriages by molecular or mechanical mechanisms [20]. The most common myomas causing RPL are submucosal myomas; they appear in 4.5% [18]. Uterine polyps can be found in 2–3% of women who cannot carry a pregnancy to term [21].

#### 5.2.2. Cervical Insufficiency

Cervical weakness is one of the causes of recurrent pregnancy loss, but it is most common in the second trimester. The diagnosis is made mainly on the basis of a history of second-trimester pregnancy loss preceded by the spontaneous rupture of amniotic membranes or cervical dilatation that is painless [1].

In 2020, there was a retrospective cohort study of all pregnancies complicated by a pregnancy loss between 14  weeks and 21  weeks + 6  days gestation. In this study, the frequency of miscarriage caused by cervical weakness was 43.4%. RPL occurred in 21.8% of cases [22].

### 5.3. Endocrine Factors

#### 5.3.1. Hyperprolactinemia

Prolactin disorders are associated with a higher rate of infertility and miscarriages. Hyperprolactinemia leads to changes in the hypothalamic–pituitary–ovarian axis, leading to impaired folliculogenesis and anovulation [2,16].

#### 5.3.2. Thyroid Disorders

Hypothyroidism is known to be one of the causes of recurrent miscarriages [23]. Overt hypothyroidism is easy to diagnose and treat, but there are contradictions regarding the relationship between subclinical hypothyroidism and pregnancy loss [16]. In a systematic review investigating whether subclinical hypothyroidism is associated with recurrent pregnancy loss, it was concluded that it is inconclusive whether patients with a history of recurrent pregnancies have a higher incidence of subclinical hypothyroidism than women not suffering from RPL [24]. However, the lack of clarity seems to be due to newer guidelines suggesting a new upper limit for the thyroid-stimulating hormone (TSH) of 4.0 mIU/L instead of the previous 2.5 mIU/L [7]. Some studies have shown a connection between the presence of antithyroid antibodies and recurrent miscarriages in euthyroid women, but there is still not enough evidence for the routine screening of antithyroid antibodies in patients with RPL [16,25,26,27].

There are studies examining the status of thyroid peroxidase antibodies (TPOAb) as a predictor of giving birth to a live child in patients with unexplained RPL. One study assessed the positivity of TPOAb, which predicted a reduced live birth rate; however, with the use of thyroxine, the chance of a live birth increased [28]. However, the T4LIFE study, which studied euthyroid women with RPL who were TPO-Ab-positive with placebo plus levothyroxine, did not show a higher live birth rate and therefore did not recommend levothyroxine in euthyroid women with RPL [29].

#### 5.3.3. Polycystic Ovary Syndrome

Polycystic ovary syndrome (PCOS) has been connected with a higher risk of pregnancy loss, but the exact mechanism is still unclear. The increased risk of miscarriage in female patients with PCOS has been recently linked to insulin resistance, hyperinsulinemia and hyperandrogenaemia [1] or increased plasminogen activator inhibitor-1 activity [16].

#### 5.3.4. Ovarian Reserve

There is still insufficient evidence for a link between the ovarian reserve and recurrent pregnancy loss.

In 2020, a study was conducted on the impact of a lower ovarian reserve on RPL. However, this study suggested that further estimations are essential for any prognostic value of performing anti-Müllerian hormone or antral follicle counts [30].

A well-established measure of ovarian reserve is the anti-Müllerian hormone (AMH), which is a homodimeric glycoprotein produced by the granulosa cells of the preantral and antral follicles. The latest analysis showed a connection between low anti-Müllerian hormone levels and the risk of pregnancy loss in patients achieving pregnancy using assisted reproductive technology [31]. However, a study examining the effect of the hormone on RPL is lacking. Until 2021, research was conducted in Denmark on the effect of the anti-Müllerian hormone concentration, which is an attractive clinical biomarker that guides infertility treatment and optimizes ovarian stimulation protocols. A study of a big number of patients with unexplained RPL showed no connection between AMH levels and subsequent live births. Therefore, AMH should not be included in the standard screening of female patients with RPL [32].

### 5.4. Male Factor

Until recently, recurrent pregnancy loss was thought to be a female-only problem. If a man was able to fertilize an egg, his sperm were considered as normal, and it was claimed that the loss of pregnancy was on the woman’s side. In 2014, research was conducted on the impact of the quality of male sperm, occupational exposure and lifestyles on the occurrence of RPL [33]. Sperm in men whose partners suffered from RPL had significantly reduced viability, reduced sperm with a normal morphology, reduced total progressive mobility and a higher mean percentage of damaged DNA than men whose partners did not suffer from RPL. In 2020, one study found that a higher sperm DNA fragmentation index (DFI) was connected with RPL, but there is still not enough evidence [34].

### 5.5. Immunological Factors

#### 5.5.1. Antiphospholipid Syndrome

Antiphospholipid syndrome (APS) is the most important and curable reason for RPL. This concerns the association between antiphospholipid antibodies—lupus anticoagulant (LA), anticardiolipin antibodies (aCL) and anti-B2 lycoprotein-I—and adverse pregnancy outcomes or vascular thrombosis. About 5 to 20% of patients suffering from RPL have positive antiphospholipid antibodies [2]. The guidelines for APS diagnostics are presented in Table 2 [35]. APS can be defined as primary in patients without underlying disease and secondary when it is associated with other conditions. Antiphospholipid antibodies are also connected with other obstetrician complications, such as preeclampsia, intrauterine growth retardation and prematurity. The target of antiphospholipid antibodies is the trophoblast. It caused an impaired trophoblastic invasion and the faulty secretion of hCG and the growth factor. It induces syncytiotrophoblast apoptosis and an inflammatory response through the activation of a complement at the maternal–fetal interface [36]. Antibodies lead to the formation of abnormal vessels such as spiral arteries [37]. In female patients suffering from RPL connected with antiphospholipid antibodies, the healthy birth percentage in pregnancies without pharmacological intervention has been about 10% [1].

One of the histological systematic reviews has shown that it is not clear if thrombosis is the primary cause of miscarriage during pregnancy in antiphospholipid syndrome. This study notes that the placenta of female patients with antiphospholipid antibodies usually shows evidence of intravascular or intervillous blood clots. This evidence indicates that these antibodies might have an influence on the viability, syncytialization and invasion of trophoblasts via β2GPI expressed on the surface of them. Moreover, in one study, inflammation in the maternal decidua and complement deposition in the placentas of patients who suffer from APS also showed antibodies-induced complement activation and proinflammatory cytokine production by trophoblasts and the neutrophils and monocytes surrounding trophoblasts at the mother and fetus connection [38].

#### 5.5.2. Inherited Thrombophilia

Thrombophilia is a group of disorders that affect a blood hypercoagulable state and induce thrombosis, which can be widely found in RPL. Thrombophilia is one of reasons for RPL, with the supposed mechanism being thrombosis of the uteroplacental circulation. The factors of thrombophilia can be prothrombin gene (PT G20210A) mutation, protein C and protein S deficiency (PSD), antithrombin III (ATIII) deficiency and methyltet-rahydrofolate reductase (MTHFR) mutation [39]. The most common is factor V Leiden mutation (FVL). There is more and more research about thrombophilia, but the connection between thrombophilia and RPL is still unclear. Originally, the hemostatic system adapting to a hypercoagulable state in pregnant patients was a means of protection, but hypercoagulation can lead both the mother and child to complications such as pre-eclampsia, placental abruption, fetal growth restriction and RPL [40].

One of the studies of women with RPL indicates that the mechanism of miscarriages is thrombosis of the temporal vessels, which impairs the blood supply to the fetus [41,42].

A 2003 meta-analysis of 31 studies found prothrombin gene mutation, V Leiden mutation and protein S deficiency to be connected with RPL [43]. One early retrospective work of research found various levels of correlation. However, prospective studies failed to confirm the association [16,44]. However, a connection between hereditary thrombophilia and RPL has been suggested [45], but prospective cohort studies that have been recently conducted have failed to confirm an association [44,46]. The routine testing of female patients suffering from RPL for inherited thrombophilia is not currently suggested [47].

#### 5.5.3. Other Immunological Factors

There is unclear evidence to support the hypothesis of human leukocyte antigen incompatibility in couples who suffer from RPL, a lack of maternal leucocytotoxic antibodies or a lack of maternal blocking antibodies.

There were studies about natural killer (NK) cells indicating that peripheral blood NK cells are phenotypically and functionally different from uterine NK cells [48]. Moreover, there is not enough evidence that changed NK cells are related to recurrent pregnancy loss. Due to this, the routine investigation of couples with RPL is not recommended. It has been suggested that uterinal NK cells may have a significant role in trophoblastic invasion and angiogenesis in addition to being an important component of the local maternal immune response to pathogens [49]. Attention should be paid to the one big study checking the connection between uterine NK cell numbers and future pregnancy outcomes, which reported that raised uterine NK cell numbers in patients with RPL was not connected with an increased risk of miscarriage [50,51]. In 2021, a study on surface NK cell markers was conducted to recognize the baseline inflammatory profile in female patients with RPL and recurrent implantation failure. The study consisted of measuring the expression of surface markers such as NKp30, TIGIT, NKp46 and DNAM-1 on complete peripheral blood NK subsets and regulatory and cytotoxic NK cells. It was found that the expression of NKp30 and TIGIT on cytotoxic NKs was significantly higher in women suffering from RPL, which may indicate their involvement in the process of miscarriage [52].

There is no unequivocal evidence of the impact of antinuclear antibodies (ANA) on RPL: at the moment, no international guidelines recommend routine ANA testing; only the latest ESHRE guidelines include testing for explanatory purposes [3,4,53]. There are a few possible actions in which ANA may play a role in the pathogenesis of RPL:

- reduced oocyte quality and embryo development [54],
- activation of the intraplacental complement cascade [55],
- deposition of immune complexes in placental tissue [55,56],
- activation of plasmacytoid dendritic cells resulting in an increased production of inflammatory cytokines [57].

International guidelines state that if higher ANA titers are found in RPL couples, the antibodies should be further differentiated to rule out Sjögren’s syndrome or lupus erythematosus.

Recently, the combination of the HLA-DRB1*03 phenotype and plasma mannose-binding lectin has been shown to predispose to the formation of autoantibodies in women with RPL. It has been proven that a series of autoantibodies can be detected in women with RPL and may negatively affect the prognosis of pregnancy. One study showed that patients with plasma mannose-binding lectin ≤500 µg/L had slightly higher rates of autoantibodies, especially ANA. The discovery that no HLA-DRB1 allele other than HLA-DRB1*03 seems to interact with low mannose-binding lectin levels is a new discovery. However, new independent studies are needed to confirm these associations [58].

In 2021, a study was conducted to determine whether the measurement of various specific markers of extracellular vesicles in plasma before and during pregnancy can be used as a predictor of pregnancy loss in female patients with RPL. These vesicles are involved in many different physiological processes, and their contents reveal the origin of cellular and pathophysiological states in various conditions [59]. In total, 17 markers were tested, and 1 marker of extracellular vesicles, CD9, was found to show a significant increase from pre-pregnancy to 6 weeks of gestation in a group of women with pregnancy loss. Changes in the remaining 16 markers were insignificant. When used in addition to intravenous immunoglobulin (IVIG), the results of this research showed an overall increase in all 17 markers of extracellular vesicles in women with RPL and 15 markers with a substantial increase. Future larger studies should investigate whether the increase in CD9-positive electric vesicles characterizes patients with RPL who have miscarried [60].

#### 5.5.4. HIV

Pregnancy loss connected with human immunodeficiency virus (HIV) infection is poorly understood, but HIV can be a risk factor. There are several works of research that have estimated pregnancy loss in patients suffering from HIV in the era of potent combination antiretroviral therapy. One study hypothesized that maternal HIV infection leads to an increased risk of pregnancy loss, including both miscarriage and stillbirth. However, determining the etiology of pregnancy loss in the case of HIV infection is difficult due to the possibility of the coexistence of genetic abnormalities and coexisting systemic diseases, such as endocrine, infectious, autoimmune and hematological factors. In HIV-infected women, pregnancy loss is a known adverse effect associated with an unsuppressed viral load; the viral load may be a significant determinant of this risk in a dose-dependent manner [61]. Research conducted in 2015 in the USA showed that a pregnant woman’s viral load, as measured by plasma HIV RNA, predicted pregnancy loss significantly better than a lifetime cumulative viral load [62]. Although the etiology of pregnancy loss has many factors, altered immune activation and prolonged inflammation, which are induced by HIV infection, are significant by interfering with decidual maintenance and contributing to placental dysfunction [61].

### 5.6. Metabolic Factors

#### 5.6.1. Acute Intermittent Porphyria (AIP)

One study showed an association between uncontrolled acute intermittent porphyria and spontaneous abortion. Acute intermittent porphyria is a rare autosomal dominant metabolic disease that is caused by a mutation in the gene for the porphobilinogen deaminase enzyme in the heme biosynthesis. This mutation causes the increased excretion of the porphyrin precursors. The attack of disease can be triggered by many different factors such as drugs, pregnancy, menstruation, hormones, changes in diet, alcohol, infectious diseases and others. Pregnancy is a significant risk factor for exacerbations in patients with acute intermittent porphyria. However, the connection between pregnancy and AIP as a cause of spontaneous pregnancy loss is rare [63,64].

#### 5.6.2. Plasma Mannose-Binding Lectin

Many studies are currently underway to determine whether plasma mannose-binding lectin levels are related to RPL. Mannose-binding lectin is an important element of the innate immune system. Low levels of mannose-binding lectin have been connected with recurrent pregnancy loss, while the association with increased levels has been poorly checked. A recent study found that the prevalence of low levels of mannose-binding lectin was significantly higher in women with RPL, while high levels were much less prevalent. No relationship was discovered between lectin levels and reproductive and perinatal outcomes before and after pregnancy loss [65].

### 5.7. Lifestyle

It has been suggested that nicotine addiction has an adverse effect on trophoblastic function and is associated with an increased risk of intermittent pregnancy loss [66].

A weight/BMI above 25 has also been shown to be connected with a higher risk of recurrent pregnancy loss in patients who conceive naturally [67].

Still, there are not enough studies to confirm other lifestyle habits as among the reasons for RPL. Addiction to cocaine use, alcohol abuse (three to five drinks per week) and higher caffeine consumption (>three cups of coffee) have been associated with a risk of pregnancy loss [68].

## 6. Diagnostics

It is possible to find genetic causes of recurrent pregnancy loss due to the analysis of the pregnancy or fetal tissue. In published studies, different genetic techniques were used, such as conventional karyotyping, fluorescence in situ hybridization (FISH) and array-based comparative hybridization (array-CGH). Analysis by conventional karyotyping is restricted by the failure of tissue culture and the fact that it does not differentiate maternal contamination from a normal female fetus [69]. FISH is limited because of the used probes, which use only some chromosomes. That means it can not detect chromosomal causes of miscarriage. Array-CGH is actually the most common technique and the most preferred. This technique checks all chromosomes and avoids limits connected with karyotyping and FISH [70,71]. New techniques, such as next-generation sequencing (NGS), are still being researched and may be used in the future [72].

Due to the higher frequency of RPL caused by uterine malformations, there is a need for uterine imaging [73]. Imaging of uterine defects has many different techniques, each with different advantages and disadvantages in different types of malformations [74]. Currently, the gold standard for the diagnosis of uterine defects is hysteroscopy and laparoscopy, due to its direct visualization. The main imperfection of this technique is the invasive nature of the procedure [75]. Another diagnostic technique is sonohysterography (SGH), which seems to be a safe method. It is said to be more informative than hysterosalpingography (HSG) and ultrasound. SGH has a higher sensitivity and specificity than HSG and diagnostic hysteroscopy [76]. 3D ultrasound can be used for diagnosis, as it allows for visualization of the internal and external contours of the uterus, is characterized by high sensitivity and specificity and is non-invasive [75,77]. Several studies have explored the usefulness of MRI but have not defined it for detecting uterine abnormalities. MRI allows for the simultaneous assessment of the uterine cavity and fundus [78]. So far, studies have not recommended testing in the case of suspected adenomyosis in patients with RPL; however, the latest update of the ESHRE guidelines recommends that all women undergo a 2D ultrasound to exclude adenomyosis [4].

One of the key issues in RPL is prolactin, so it is important to get its level checked. It should be tested in the follicular phase. Studies have found that both elevated and decreased levels of prolactin can contribute to RPL [79,80].

In women suffering from RPL due to thyroid defects, it is very important to check the levels of hormones and antibodies such as thyroid peroxidase antibodies (TPO-Ab), thyroid autoantibodies (TGAb), anti-TSH receptor antibodies (TSHr-Ab), triiodothyronine (T3), thyroxine (T4) and TSH. The studies should take into account the presence of subclinical hypothyroidism with a TSH level >2.5 mIU/L and triiodothyronine and thyroxine levels within normal limits [3,4].

Polycystic ovarian syndrome is connected with insulin resistance, which means that when this syndrome is suspected, the parameters of insulin metabolism should be tested. Some studies have assessed several markers such as fasting insulin (FI), fasting glucose (FG), the fasting-glucose-to-insulin ratio (FG/IF) and insulin resistance (IR). In patients with suspected PCOS, the HOMA-IR insulin resistance index can also be determined, which is a quantitative assessment of the contribution of insulin resistance and impaired β-cell function to fasting hypoglycemia. The HOMA-IR index is significantly higher in patients with RPL [3,4].

In order to test the ovarian reserve in patients with suspected RPL, the levels of FSH, estrogens, inhibin B and anti-Müllerian hormone should be assessed; the examination should also include ultrasound to assess the antral follicle count and the volume of the ovaries.

Not only are female factors risk factors for recurrent miscarriages. Semen should also be tested. Sperm should be tested for viability, morphology and total progressive sperm motility. A risk factor is the higher average percentage of sperm with damaged DNA. In addition, exposure to harmful factors and the lifestyle of men should be taken into account [3]. So far, the guidelines have suggested assessing partners’ lifestyle factors (smoking, alcohol consumption, exercise patterns and body weight). In contrast, the updated guidelines recommend examining paternal age, smoking, alcohol consumption, exercise patterns and body weight [4].

Another factor of RPL is acquired thrombophilia—antiphospholipid syndrome (APS). There are some antibodies associated with thrombosis such as lupus anticoagulant (LA), anticardiolipin antibodies (ACA) and β2glicoprotein I antibodies. In diagnostics, the criteria included in Table 1 should be used. There was a cohort study which reported that it is justifiable to offer testing for antiphospholipid syndrome to all patients with two or more miscarriages in the past [8]. For hereditary thrombophilia, women with RPL may be tested for factor V Leiden mutations, prothrombin mutations, protein C mutations, protein S mutations and antithrombin deficiency. However, screening for hereditary thrombophilia is not recommended in women with a history of RPL due to the poor correlation. Testing may be carried out if there are additional risk factors for hereditary thrombophilia—for example, a history of thromboembolism or hereditary thrombophilia in a family member. Due to the changes that occur in the pregnant woman’s body, the proper interpretation of the results and the diagnosis of hereditary thrombophilia are possible in the case of factor V Leiden and prothrombin mutations, but it can be problematic in the case of antithrombin, protein C and protein S. Therefore, it is important that tests lead in this direction after 6 weeks from the loss of pregnancy [81,82].

The diagnosis of RPL should also include testing for immunological factors. Considering the HLA antigen, testing for HLA allele compatibility between partners to reduce the likelihood of developing “blocking antibodies” that protect against fetal miscarriage, testing for the frequency of HLA alleles in women with recurrent miscarriages and testing for HLA-C and HLA-G alleles should be considered. Studies involving the study of the effects of anti-HY antibodies, cytokines, antinuclear antibodies, NK cells and other immunoassays have also been conducted, but there is still insufficient evidence for the usefulness of these studies [3]. According to the updated ESHRE guidelines, HLA testing in women with RPL is not recommended in clinical practice. However, the determination of HLA class II in Scandinavian women with secondary RPL after giving birth to a boy may be considered for prognostic purposes [4].

If acute intermittent porphyria is suspected, laboratory testing including porphyrin derivatives and porphobilinogen should be performed. Their increase may suggest the presence of the disease [63].

Diagnostics of HIV infection, which is one of the factors of miscarriage, include rapid HIV tests implemented in many institutions [61]. Pregnant patients need to be screened early in pregnancy. The standard-of-care test for diagnosing HIV in a clinical setting is the serum test, known as the HIV 4th generation test, which combines antibody (Ab) and antigen (Ag) tests [83].

## 7. Treatment

### 7.1. Chromosomal Factors

Some of methods used in the management of recurrent pregnancy loss caused by genetic factors are Preimplantation Genetic Diagnosis (PGD) and Preimplantation Genetic Screening (PGS). There is increasing attention being paid to the possible utility of assisted reproductive technology (ART) with or without PGD or PGS in the treatment of RPL. Two related techniques are being used for the genetic analysis of embryos created through ART. PGD tests are used for specific genetic abnormalities which are known to be heritable and present in one or both of the parents. PGS uses a more global genetic assessment of the embryo to detect a wide variety of genetic abnormalities. PGS is more likely to be used in couples with spontaneous RPL. Both approaches allow for the selection of embryos that can be transferred, which is based on genetic criteria. Affected embryos are generally not transferred back into the uterus. PGD is mainly exploited in carrier couples with no history of infertility or RPL. Its use in RPL couples is mostly limited to translocation carriers, because most disorders that affect one gene do not result in sporadic or RPL. PGS involves the analysis of all twenty-three chromosome pairs, with several molecular techniques used across the years, including FISH, CGH, array CGH (aCGH), single-nucleotide polymorphism array, quantitative or real-time polymerase chain reaction and next-generation sequencing, also known as massive parallel sequencing [15,16].

The finding of an abnormal parental karyotype should persuade a couple to visit a clinical geneticist. Genetic counseling offers the couple a prognosis for the risk of future pregnancies complicated with an unbalanced chromosome complement and the opportunity for familial chromosome studies. Preimplantation genetic diagnosis has been suggested as a treatment method for translocation carriers [1].

ASRM’s guidelines take genetic counseling as the first method of dealing with recurrent pregnancy loss. It says that management options include PGD for specific translocations, with the transfer of unaffected embryos or the use of donor gametes [2].

The ESHRE guidelines identify PGT as the main treatment method. Embryos are screened for chromosomal abnormalities before implantation. Data from published studies are limited due to the preimplantation genetic treatment method used, as the vast majority of cases are carried out using FISH [3,4].

Each of the guidelines state that couples suffering from recurrent pregnancy loss should be informed that they have a greater (50–74%) chance of having a healthy, viable child in future pregnancies after natural conception than what is currently achieved with preimplantation genetic diagnosis/in vitro fertilization (30–35%) [1,2,3,4].

### 7.2. Anatomical Factors

#### 7.2.1. Uterine Anomalies

RCOG guidelines say that there is not enough evidence to evaluate the effect of uterine septum removal in patients with recurrent pregnancy loss and uterine septum to prevent miscarriage in the future. Still, there are no published randomized trials assessing the benefits of the surgical correction of uterine abnormalities on the pregnancy outcome [1].

The treatment of anatomical uterine defects, according to the ASRM guidelines, takes into account the occurrence of Asherman syndrome/intrauterine synechiae, uterine fibroids and uterine polyps, which is controversial, and there is no conclusive evidence that surgical management reduces the risk of miscarriage. According to the absence of randomized trials, there is general agreement that the surgical correction of significant uterine defects should be considered. In the case of irreversible anatomical defects of the uterus and RPL, in vitro fertilization with the transfer of embryos to a properly selected carrier may also be a clinical consideration [2].

The ESHRE guideline is very extensive about possible treatments for anatomical defects. In cases of a septate uterus, hysteroscopic metroplasty has become the treatment of choice. The hysteroscopic treatment of a symptomatic uterus with a septum can be accomplished by using many different methods, including hysteroscopic scissors and electrosurgical electrodes fitted through the hysteroscope or resectoscope. There is no evidence of which method gives better results. For hemi-uteruses, uterine reconstruction is not possible. But in cases of hemi-uteruses with rudimentary horns and cavities, the laparoscopic resection of the rudimentary horn should be considered to avoid ectopic pregnancy in this cavity. In cases of bicornuate uteruses, the method of choice should be metroplasty (transabdominal or laparoscopically). Overall, there is no strong evidence that metroplasty in women with RPL and a bicornuate uterus allows for to-term pregnancy and a live birth. The clinical treatment of women with RPL with endometrial polyps, submucosal or intramural fibroids is still controversial. There is not enough evidence that polyps or fibroids are connected with a higher risk of pregnancy loss, and there is not enough evidence that surgical management reduces the risk. Moreover, hysteroscopic myomectomy in the treatment of fibroids can be associated with postoperative complications that can affect future pregnancies, including the formation of intrauterine adhesions and the risk of uterine rupture during pregnancy. ESHRE states that there is no strong evidence of a benefit for the surgical resection of intrauterine adhesions for pregnancy outcomes. After the hysteroscopic resection of intrauterine adhesions in patients with recurrent pregnancy loss, precautions have to be taken to prevent the recurrence of adhesions [3,4].

#### 7.2.2. Cervical Insufficiency

In cases of cervical weakness, a method of choice is cervical cerclage. In RCOG’s guidelines, it is said that cervical cerclage is associated with a high risk of miscarriage, and it is related to the surgery and the risk of stimulating uterine contractions. That is why it should be considered only in female patients who are likely to benefit. Transabdominal cerclage can be recommended as a treatment for second-trimester miscarriage and for the prevention of early preterm labor in patients with a previous failed transvaginal cerclage and/or a very short and scarred cervix [1].

ESHRE’s position on RPL states that cervical cerclage has been used in the prevention of preterm birth in female patients with a second trimester pregnancy loss in the past or risk factors such as a short cervix revealed at USG. But there was a study conducted by Cochrane on the topic of using a cervical cerclage for preventing pregnancy loss, which found not enough evidence that prophylactic cerclage lowers the risk of pregnancy loss or preterm delivery in females at risk of preterm birth or mid-trimester loss due to cervical weakness [3,4,84].

### 7.3. Endocrine Factors

RCOG guidelines state that there is not enough evidence to prove that the treatment of endocrine disorders results in higher levels of live birth rates [1].

A different position is taken by the ASRM guidelines, which agree that maternal endocrine disorders like diabetes and thyroid dysfunction should be diagnosed and treated. It is recommended to check prolactin levels because of their influence on ovulatory dysfunction. High levels of the prolactin hormone can be associated with RPL through alterations in the hypothalamic–pituitary–ovarian axis, resulting in impaired folliculogenesis and oocyte maturation and/or a short luteal phase. The normalization of prolactin levels with a dopamine agonist, like bromocriptine, improved subsequent pregnancy outcomes in women with RPL [85]. There were some studies checking the influence of the administration of progestogen, but to patients with sporadic pregnancy loss, the influence is ineffective. However, in women with three or more consecutive pregnancy losses immediately preceding their current pregnancy, empiric progestogen administration can be of some potential benefit [2].

ESHRE addresses the topic of hypothyroidism in women suffering from RPL. Hypothyroidism in pregnant women is connected with adverse pregnancy complications like a higher risk of premature birth, a low birth weight and miscarriages in the future as well as detrimental results on fetal neurocognitive development. The European Thyroid Association Guidelines for the Management of Subclinical Hypothyroidism in Pregnancy states that pregnant patients should be treated with levothyroxine. Also, the American Thyroid Association recommends levothyroxine treatment for pregnant women suffering from RPL with subclinical hypothyroidism and recommends considering treatment for pregnant women with TSH concentrations >2.5 mU/L and TPOAb or TSH >10.0 mU/L [3,4].

In cases of women with insulin resistance and PCO syndrome, metformin is a low-risk and effective oral hypoglycemic agent. In patients with PCOS, metformin significantly reduces the rate of pregnancy loss. The latest meta-analysis regarding the risks of metformin during pregnancy concluded that exposure to metformin during the first trimester of pregnancy does not increase the risk of birth complications [86]. Some proof may support the use of metformin treatment to increase the live birth rate in female patients with PCO syndrome, but in the absence of any substantial research on patients with RPL and PCOS, metformin is not suggested [3,4].

The ESHRE guidelines also give information about the treatment of hyperprolactinemia in women with RPL. Prolactin testing is only suggested in patients with RPL if they have clinical symptoms indicative of hyperprolactinemia. Women with high levels of the prolactin hormone who require medical management are typically treated with dopamine agonist therapy like bromocriptine or cabergoline. Some studies state that using bromocriptine may be considered in patients with RPL and hyperprolactinemia to increase the live birth rate [3,4].

### 7.4. Male Factor and Lifestyle

There is moderate evidence of a connection between sperm DNA quality and pregnancy loss. Moreover, the quality of sperm is associated with unhealthy lifestyles and disease [3].

One of the factors affecting the quality of male sperm is smoking. The connection between smoking cigarettes and reduced male fertility, heritable genomic damage and the incidence of childhood neoplasms and the impaired mental health of offspring has been well discovered. But there is still not enough evidence on whether paternal smoking discontinuation has a positive effect on RPL [3,4].

Obesity is connected with impaired sperm parameters and sperm DNA damage. But there is still not enough evidence proving that paternal weight loss has an impact on the live birth rate or recurrent pregnancy loss [3,4].

Another important factor of the bad quality of sperm is a long list of used medications. For example, selective serotonin reuptake inhibitors, corticosteroids, antibiotics and anti-inflammatories may destroy sperm function [3,4].

Diet can also have an influence on the quality of male sperm. A well-balanced diet, rich in carbohydrates, fiber, vegetable protein and water, is connected with a good quality of sperm, like good motility, morphology and DNA quality. Restricting the intake of fats, especially trans-fats, and sugars is also connected with healthy sperm. Natural antioxidants in the form of vitamins E and C and microelements like selenium, iron and zinc give higher levels of reactive oxygen species [3,4].

There was a review of 34 research works which reported that men with a bad sperm quality showed improvement in sperm parameters following antioxidant treatment in the Cochrane Database in 2014. Despite this, there is still too little evidence for this to be definitive, and further research is needed [87].

### 7.5. Immunological Factors

#### 7.5.1. Antiphospholipid Syndrome

Pregnant women with antiphospholipid syndrome should be considered for management with low-dose aspirin plus heparin to prevent pregnancy loss in the future. One meta-analysis demonstrates that the only treatment or treatment combination that leads to a substantial increase in the live birth rate among patients with APS is aspirin plus unfractionated heparin. This connection of substances substantially reduces the risk of pregnancy loss by 54% [88]. Pregnancies complicated with APS treated with aspirin and heparin remain at a high risk of complications during the whole pregnancy. Although aspirin connected with heparin treatment substantially increases the live birth rate of patients with RPL associated with antiphospholipid antibodies, these pregnancies remain at a high risk of complications during the whole pregnancy, including RPL, pre-eclampsia, fetal growth restriction and preterm birth. For thrombosis prophylaxis, low-molecular-weight heparin is better than unfractionated heparin because of a lower risk of osteoporosis and heparin-induced thrombocytopenia [1,2,3,4]. The recommendations of the ESHRE guidelines note that, for female patients who fulfill the laboratory criteria of APS and have a history of three or more pregnancy losses, we recommend administration with low-dose aspirin (75 to 100 mg/day) starting before conception and a prophylactic dose of heparin (UFH or LMWH) starting at the date of a positive pregnancy test over no treatment [3,4].

In 2019, a study was published on the use of granulocyte colony-stimulating factor (G-CSF), particularly in the case of a previous pregnancy loss not related to chromosomal aberrations. This study showed that G-CSF increased the number of regulatory T cells in RPL patients, as well as the expression of G-CSF and VEGF in villous trophoblasts, which may indicate its therapeutic efficacy by regulating the maternal immune response, recruiting regulatory T cells in the decidua and increasing the number of trophoblasts [89].

Due to the finding of dubious evidence regarding the impact of natural killers and cytokines on RPL, it is questionable to select woman for specific therapies due to the presence or absence of specific immunological biomarkers.

Several studies have tested the use of IVIG with various autoantibodies or NK cell/cytokine aberrations, but these studies were unrepresentative.

The latest ESHRE guidelines state that no immunological biomarker other than high-titer antiphospholipid antibodies may be used to select couples with RPL for specific therapy [4].

#### 7.5.2. Inherited Thrombophilia

Guidelines state that there is no strong evidence for evaluating the result of heparin during pregnancy to prevent a pregnancy loss in patients with recurrent first-trimester miscarriage connected with inherited thrombophilia. Female patients with known heritable thrombophilia are at a high risk of venous thromboembolism. There is a prospective randomized trial which demonstrates the efficiency of the low-molecular-weight heparin–enoxaparin for the management of females with a history of a single late pregnancy loss after 10 weeks of gestation who carry the factor V Leiden or prothrombin gene mutation or have protein S deficiency. However, none of the guidelines do not recommend using antithrombotic prophylaxis [1,3,4].

#### 7.5.3. Other Immunological Factors

One systematic review showed that the use of many different forms of immunotherapy, including immunization with paternal cells, third-donor leukocytes, trophoblast membranes and intravenous immunoglobulins, in patients with unexplained RPL had no relevant benefit in preventing further miscarriage compared to placebo [90]. In 2010, a meta-analysis of the effectiveness of IVIG treatment was performed. The study involved the administration of IVIG and saline under controlled conditions to women with idiopathic secondary recurrent pregnancy loss. A total of 82 patients were examined, of whom 47 became pregnant. All pregnancies ended in a live birth. This meta-analysis showed no benefit of IVIG in the management of RPL. Despite the lack of effectiveness of IVIG in the treatment of RPL, IVIG susceptibility has been found to significantly increase the live birth rate after the initiation of therapy before conception and has a borderline significant therapeutic result in patients with secondary RPL [91]. Recently, a high-quality randomized trial showed that the intravenous administration of repeated doses of immunoglobulin (400 mg/kg) for five consecutive days in very early pregnancy to female patients with four or more RPLs significantly increased the live birth rate [92]. It turns out that the study is important enough to correct the recommendations and suggest the use of IVIG in the treatment of women with RPL [4]. However, immunotherapy is costly and potentially associated with serious side effects, including transfusion reactions, anaphylactic shock and hepatitis [93]. There is no published information on the use of anti-TNF drugs to improve pregnancy outcomes in women with RPL. Treatment with IVIG has also been suggested for unexplained pregnancy loss. However, some studies and meta-analyses have concluded that intravenous immunoglobulin therapy has low effectiveness in primary RPL and is therefore not recommended [1,2,3,4].

#### 7.5.4. HIV

Antiretroviral therapy (ART) is used in HIV-infected women. The provision of ART to women before and during pregnancy and breastfeeding prevents mother-to-child transmission and improves the health and survival of women, which itself benefits the health of their newborns [94,95]. Pregnancy became the first circumstance in which management used as prevention was applied programmatically, after the publication of the results of randomized clinical trials. Without any maternal antiretroviral therapy, about 15–30% of formula-fed children and up to 45% of those who are breastfed will become infected with HIV [96]. WHO guidelines regarding HIV infection currently suggest a fixed-dose combination of tenofovir disoproxil fumarate with either lamivudine or emtricitabine plus the non-nucleoside reverse transcriptase inhibitor (NNRTI) efavirenz as a first-line treatment [94,95]. Nowadays, a lot of pregnant patients with HIV in developed countries receive ART at the time of conception; guidelines advise patients to review their ART regimens, but it is generally suggested that pregnant patients should continue their previous treatment if there are no tolerance issues and viral loads are lowering [96].

### 7.6. Metabolic Factors

#### Acute Intermittent Porphyria

Spontaneous abortions are most likely caused by uncontrolled AIP. Attacks appear mostly during the first trimester of pregnancy. There is only limited research supporting the use of hemin during pregnancy. An acute attack during pregnancy seems not to have a detrimental effect on the child. It is said that women suffering from acute intermittent porphyria are related to higher rates of morbidity and complications during pregnancy, but accurate monitoring throughout the porphyric pregnancy could ensure a good outcome. Still, there is no consistent guidance available for pregnancy. Experts maintain that asymptomatic patients suffering from AIP should start with pregnancy without any restriction. In women with symptoms, it is recommended to avoid getting pregnant after 1 year from the last attack. The prevention of pregnancy loss in patients with AIP is still controversial and should be further investigated [63].

## 8. Unexplained Pregnancy Loss

Patients with unexplained RPL have a good prognosis for future pregnancies without medication if they are provided with supportive care in a dedicated early pregnancy assessment unit. A significant proportion of RPL cases remain unexplained despite a detailed examination. These patients may be informed that the prognosis for a successful live birth in the future with supportive care is only in the region of 75%. One of methods used in unexplained pregnancy loss is aspirin alone or in combination with heparin [1,2].

## 9. Perspectives in the Diagnosis and Treatment of Recurrent Pregnancy Loss

So far, some problems have been solved; factors such as uterine anomalies, cervical insufficiency and endocrine factors have already been extensively studied, and we know their treatment methods, but many issues still remain to be explored.

Low-molecular-weight heparin is currently being trialed in women with RPL and hereditary thrombophilia. This is a study called ALIFE2. The aim of the ALIFE2 study is to evaluate the efficiency of low-molecular-weight heparin (LMWH) in female patients with hereditary thrombophilia and RPL, with live delivery as the primary outcome. The study population is pregnant women with less than 7 weeks of gestation, confirmed inherited thrombophilia and a history of two or more pregnancy losses or intrauterine fetal deaths or both. The results of the ALIFE2 study will clarify the need for screening for inherited thrombophilia in patients with RPL. The efficiency of LMWH in patients with RPL and hereditary thrombophilia has never been tested in a randomized controlled trial. Thus, if the results of the ALIFE2 study show that LMWH increases the likelihood of live birth in women with RPL and inherited thrombophilia, screening for inherited thrombophilia in this setting can be warranted. On the other hand, unless evidence of a benefit is discovered, the use of LMWH will not be warranted, and screening for hereditary thrombophilia will not be recommended. This will reduce the cost of inappropriate screening and reduce the anticoagulant burden for pregnant women. This study brings hope both to patients struggling with thrombophilia and RPL and to clinicians [97].

Recently, considerable attention has been given to tacrolimus in the treatment of reproductive failures such as unexplained infertility, repeated implantation failure and RPL. Tacrolimus is an antibiotic from the macrolide group—a calcineurin inhibitor that is being used as an immunosuppressant in organ transplantation and in patients with autoimmune diseases such as rheumatoid arthritis, lupus nephritis and refractory ulcerative colitis. The enhanced immune response from T-helper cells is considered the main therapeutic aim for tacrolimus in female patients with reproductive disorders related to maternal and fetal immune abnormalities [98]. In 2015, it was reported for the first time that immunosuppressive treatment with tacrolimus improved pregnancy and embryo implantation rates in patients. In 2017, a successful pregnancy was also reported in a patient treated with tacrolimus who had a history of twelve consecutive pregnancy losses despite prior treatment with low-dose aspirin, unfractionated heparin, prednisolone and intravenous immunoglobulin [99]. Given the favorable outcomes and safety during pregnancy, tacrolimus is a promising therapeutic option for managing reproductive failure associated with maternal–fetal immune abnormalities. There is currently no consensus on the management of tacrolimus before and during pregnancy [98]. The possibility of using tacrolimus in the treatment of RPL still needs more research.

Many studies have been conducted on the relationship between celiac disease (CD) and RPL. The presence of clinical symptoms during pregnancy and the postpartum period, such as iron deficiency anemia, some episodes of diarrhea and mouth ulcers, should alert the physician to consider celiac disease. During pregnancy, this disease can cause an increased deficiency of folic acid in the blood, vitamin B12 and subsequent nutritional disorders [100]. There are conflicting data regarding the association of CD with RPL. One study compared serum celiac markers in women with and without RPL. Maternal lots were analyzed for immunoglobulin A (IgA) and immunoglobulin G (IgG) tissue transglutaminase antibodies and endomyse (EM) antibodies. These studies were conducted on a large group of women of similar ages, races, ethnicities and BMI scores. The percentage of female patients with antibodies indicative of CD was similar in female patients with RPL and in female patients with uncomplicated live births [101]. In 2019, the issue of CD and RPL was addressed again. It was re-established that the results show no substantial dissimilarity in the incidence of CD autoantibodies when comparing RPL women with controls [102]. However, a recent study from Italy that re-examined the association of celiac disease with RPL indicates that patients with RPL have an almost sixfold increased risk of CD impact compared to the rest of the population. Human leukocyte antigen (HLA) DQ2/DQ8 haplotypes are major determinants of genetic susceptibility to celiac disease. The study observed a higher incidence of HLA-DQ2/DQ8 positivity in females with idiopathic RPL compared to the general population. The authors of this study suggest that HLA-DQ2/DQ8 positivity, in the presence of exogenous stimuli, can promote deleterious inflammation in the early stages of pregnancy and thus increase the likelihood of pregnancy loss [103]. In conclusion, it is recommended to raise awareness among the medical population for celiac disease research; studies should be carried out on larger groups of patients to enable a new rational approach to diagnosis and personalized medicine.

Clinical trials on RPL are still being conducted. Currently, the use of hydroxychloroquine, which has anticoagulant, vasoprotective, immunomodulatory, glucose tolerance-improving, lipid-lowering and anti-infective properties, is being tested. This study may establish support for a new treatment option for unexplained RPL [104].

Another study is underway on the female reproductive tract microbiome, which can reflect women’s reproductive health and can be related to pregnancy outcomes. Disturbances in this microbiome can be connected with adverse reproductive outcomes. Researchers hypothesize that the composition of the endometrial and vaginal microbiome in female patients with a history of RPL is different compared to that of normal fertile women [105].

In the future, these studies may allow for determining new diagnostic paths and therapeutic options in the treatment of RPL.

However, cases of unexplained RPL remain a major problem of RPL. Therefore, further research is needed to identify additional risk factors that may contribute.

There is also a growing interest in personalized medicine, consisting in developing a personalized approach to the treatment and diagnosis of RPL. This involves tailoring interventions based on individual patient characteristics such as genetic profiles and immune system status.

Another problem concerning patients with RPL is the psychological aspect. Therefore, more research is needed to explore effective psychological support and counseling strategies for those experiencing RPL.

## 10. Conclusions

Recurrent pregnancy loss is a challenging problem which frequently appears in the OB-GYN’s office. It causes psychological stress in patients, their partners and clinicians. Couples suffering from RPL want to be aware of the reason for their pregnancy losses. The evaluation and management of RPL should be based on the reason for the loss. Realistic expectations for successful pregnancy in the future should be based on the maternal age, the number of prior losses and the results of the evaluation. Finally, awareness of the cause of pregnancy loss can have a great psychological and emotional relief, as couples frequently put the responsibility for the loss on themselves, when this is rarely the case.

Still, there is not enough evidence on the diagnostics and treatment of this issue. The criteria for referral to RPL services vary, partly due to the lack of consensus on the definition of RPL between different guidelines. Also, controversies exist regarding recommendations for investigations and the treatment of RPL. A huge majority of patients are going to give birth to a healthy live child in the next pregnancy. However, individualized approaches should be created to recognize and correct any modifiable risk factors, as well as to offer adequate psychological support to couples suffering from RPL. As before, more randomized trials on RPL and possible treatments should be conducted.

## Figures and Tables

**Table 1:** Definition of RPL and definition of pregnancy in different guidelines [1,2,4].

RCOG (2011)	ASRM (2012)	ESHRE (2023)
Three or more failed pregnancies Do not have to be consecutive	Two or more failed clinical pregnancies	Two or more failed pregnancies Do not have to be consecutive
Clinical pregnancy, also includes biochemical pregnancy	Ultrasound and histological confirmation of pregnancy	Confirmation by serum or urinary chronic gonadothropin Do not include ectopic and molar pregnancies

**Table 2:** International consensus classification criteria for the APS [35].

**International Consensus Classification Criteria for the APS** **At Least One Clinical and One Laboratory Criterion Must be Present for Defining APS**	
**Clinical criteria**	
1. Vascular thrombosis	One or more clinical episodes of an arterial, venous or small vessel thrombosis, in any tissue or organ. Thrombosis must be confirmed by imaging or Doppler studies or histopathology, with the exception of superficial venous thrombosis. For histopathologic confirmation, thrombosis should be without significant evidence of inflammation in the vessel wall.
2. Obstetric morbidity	One or more unexplained demise of a morphologically normal fetus at or beyond 10 weeks of gestation, with a normal fetal morphology documented by ultrasound or by direct examination, or One or more premature births of a morphologically normal neonate before the 34th week of gestation, because of (a) eclampsia or severe preeclampsia or (b) placental insufficiency, or Three or more unexplained consecutive miscarriages of <10 weeks of gestation. Known factors associated with recurrent miscarriage, including parental genetic, anatomic and endocrinologic factors, should be ruled out.
**Laboratory criteria**	
1. Anticardiolipin antibodies (IgG and/or IgM) in the blood, present in medium or high titers (>40 GPL or MPL or >99th percentile), on two or more occasions at least 12 weeks apart, measured by a standardized ELISA.	
2. Anti-β2GP1 antibody of IgG and/or IgM isotypes in the blood (>99th percentile) on two or more occasions at least 12 weeks apart, measured by a standardized ELISA.	
3. Lupus anticoagulant present in plasma, on two or more occasions at least 12 weeks apart, detected according to the guidelines of the International Society on Thrombosis and Hemostasis.	

## Data Availability

No new data were created or analyzed in this study. Data sharing is not applicable to this article.

## References

[1] Royal College of Obstetricians and Gynaecologists (RCOG) (2011). The Investigation and Treatment of Couples with Recurrent First-trimester and Second-Trimester Miscarriage.

[2] Practice Committee of the American Society for Reproductive Medicine (2012). Evaluation and treatment of recurrent pregnancy loss: A committee opinion. Fertil. Steril..

[3] European Society of Human Reproduction and Embryology (ESHRE) (2017). Recurrent Pregnancy Loss.

[4] Atik R.B., Christiansen O.B., Elson J., Kolte A.M., Lewis S., Middeldorp S., Mcheik S., Peramo B., Quenby S., The ESHRE Guideline Group on RPL (2023). ESHRE guideline: Recurrent pregnancy loss: An update in 2022. Hum. Reprod. Open.

[5] Wyatt P.R., Owolabi T., Meier C., Huang T. (2005). Age-specific risk of fetal loss observed in a second trimester serum screening population. Am. J. Obstet. Gynecol..

[6] Stirrat G. (1990). Recurrent miscarriage. Lancet.

[7] Chester M.R., Tirlapur A., Jayaprakasan K. (2022). Current management of recurrent pregnancy loss. Obstet. Gynaecol..

[8] Van den Boogaard E., Cohn D.M., Korevaar J.C., Dawood F., Vissenberg R., Middeldorp S., Goddijn M., Farquharson R.G. (2013). Number and sequence of preceding miscarriages and maternal age for the prediction of antiphospholipid syndrome in women with recurrent miscarriage. Fertil. Steril..

[9] Van den Boogaard E., Kaandorp S.P., Franssen M.T., Mol B.W., Leschot N.J., Wouters C.H., van der Veen F., Korevaar J.C., Goddijn M. (2010). Consecutive or non-consecutive recurrent miscarriage: Is there any difference in carrier status?. Hum. Reprod..

[10] Egerup P., Kolte A.M., Larsen E.C., Krog M., Nielsen H.S., Christiansen O.B. (2016). Recurrent pregnancy loss: What is the impact of consecutive versus non-consecutive losses?. Hum. Reprod..

[11] Delabaere A., Huchon C., Lavoue V., Lejeune V., Iraola E., Nedellec S., Gallot V., Capmas P., Beucher G., Subtil D. (2014). Standardisation de la terminologie des pertes de grossesse: Consensus d’experts du Collège national des gynécologues et obstétriciens français (CNGOF). [Definition of pregnancy losses: Standardization of terminology from the French National College of Obstetricians and Gynecologists (CNGOF)]. J. Gynecol. Obstet. Biol. Reprod..

[12] Toth B., Würfel W., Bohlmann M., Zschocke J., Rudnik-Schöneborn S., Nawroth F., Schleußner E., Rogenhofer N., Wischmann T., von Wolff M. (2018). Recurrent Miscarriage: Diagnostic and Therapeutic Procedures. Guideline of the DGGG, OEGGG and SGGG (S2k-Level, AWMF Registry Number 015/050). Geburtshilfe Frauenheilkd..

[13] Shields R., Hawkes A., Quenby S. (2020). Clinical approach to recurrent pregnancy loss. Obstet. Gynaecol. Reprod. Med..

[14] Colley E., Hamilton S., Smith P., Morgan N.V., Coomarasamy A., Allen S. (2019). Potential genetic causes of miscarriage in euploid pregnancies: A systematic review. Hum. Reprod. Updat..

[15] Hyde K.J., Schust D.J. (2015). Genetic Considerations in Recurrent Pregnancy Loss. Cold Spring Harb. Perspect. Med..

[16] El Hachem H., Crepaux V., May-Panloup P., Descamps P., Legendre G., Bouet P.-E. (2017). Recurrent pregnancy loss: Current perspectives. Int. J. Womens Health.

[17] Smits M.A., van Maarle M., Hamer G., Mastenbroek S., Goddijn M., van Wely M. (2020). Cytogenetic testing of pregnancy loss tissue: A meta-analysis. Reprod. Biomed. Online.

[18] Jaslow C.R. (2014). Uterine Factors. Obstet. Gynecol. Clin. North Am..

[19] Valle R.F., Ekpo G.E. (2013). Hysteroscopic Metroplasty for the Septate Uterus: Review and Meta-Analysis. J. Minim. Invasive Gynecol..

[20] Sinclair D.C., Mastroyannis A., Taylor H.S. (2011). Leiomyoma Simultaneously Impair Endometrial BMP-2-Mediated Decidualization and Anticoagulant Expression through Secretion of TGF-β3. J. Clin. Endocrinol. Metab..

[21] Salim S., Won H., Nesbitt-Hawes E., Campbell N., Abbott J. (2011). Diagnosis and Management of Endometrial Polyps: A Critical Review of the Literature. J. Minim. Invasive Gynecol..

[22] Joubert M., Sibiude J., Bounan S., Mandelbrot L. (2021). Mid-trimester miscarriage and subsequent pregnancy outcomes: The role of cervical insufficiency in a cohort of 175 cases. J. Matern. Fetal. Neonatal Med..

[23] Alexander E.K., Pearce E.N., Brent G.A., Brown R.S., Chen H., Dosiou C., Grobman W.A., Laurberg P., Lazarus J.H., Mandel S.J. (2017). 2017 Guidelines of the American Thyroid Association for the diagnosis and management of thyroid disease during pregnancy and the postpartum. Thyroid.

[24] Dhillon-Smith R.K., Boelaert K., Jeve Y.B., Maheshwari A., Coomarasamy A., on behalf of Royal College of Obstetricians and Gynaecologists (2022). Subclinical hypothyroidism and antithyroid autoantibodies in women with subfertility or recurrent pregnancy loss. Scientific impact paper no. 78. BJOG.

[25] De Groot L., Abalovich M., Alexander E.K., Amino N., Barbour L., Cobin R.H., Eastman C.J., Lazarus J.H., Luton D., Mandel S.J. (2012). Management of Thyroid Dysfunction during Pregnancy and Postpartum: An Endocrine Society Clinical Practice Guideline. J. Clin. Endocrinol. Metab..

[26] Bernardi L.A., Cohen R.N., Stephenson M.D. (2013). Impact of subclinical hypothyroidism in women with recurrent early pregnancy loss. Fertil. Steril..

[27] De Carolis C., Greco E., Guarino M.D., Perricone C., Lago A.D., Giacomelli R., Fontana L., Perricone R. (2004). Anti-thyroid Antibodies and Antiphospholipid Syndrome: Evidence of Reduced Fecundity and of Poor Pregnancy Outcome in Recurrent Spontaneous Aborters. Am. J. Reprod. Immunol..

[28] Bliddal S., Feldt-Rasmussen U., Rasmussen K., Kolte A.M., Hilsted L.M., Christiansen O.B., Nielsen C.H., Nielsen H.S. (2019). Thyroid Peroxidase Antibodies and Prospective Live Birth Rate: A Cohort Study of Women with Recurrent Pregnancy Loss. Thyroid.

[29] van Dijk M.M., Vissenberg R., Fliers E., Post J.A.M.V.D., van der Hoorn M.-L.P., de Weerd S., Kuchenbecker W.K., Hoek A., Sikkema J.M., Verhoeve H.R. (2022). Levothyroxine in euthyroid thyroid peroxidase antibody positive women with recurrent pregnancy loss (T4LIFE trial): A multicentre, randomised, double-blind, placebo-controlled, phase 3 trial. Lancet Diabetes Endocrinol..

[30] Bunnewell S.J., Honess E.R., Karia A.M., Keay S.D., Al Wattar B.H., Quenby S. (2020). Diminished ovarian reserve in recurrent pregnancy loss: A systematic review and meta-analysis. Fertil. Steril..

[31] Busnelli A., Somigliana E., Cirillo F., Levi-Setti P.E. (2021). Is diminished ovarian reserve a risk factor for miscarriage? Results of a systematic review and meta-analysis. Hum. Reprod. Updat..

[32] Bliddal S., Feldt-Rasmussen U., Forman J.L., Hilsted L.M., Larsen E.C., Christiansen O.B., Nielsen C.H., Kolte A.M., Nielsen H.S. (2023). Anti-Müllerian hormone and live birth in unexplained recurrent pregnancy loss. Reprod. Biomed. Online.

[33] Ruixue W., Hongli Z., Zhihong Z., Rulin D., Dongfeng G., Ruizhi L. (2013). The impact of semen quality, occupational exposure to environmental factors and lifestyle on recurrent pregnancy loss. J. Assist. Reprod. Genet..

[34] Yifu P., Lei Y., Shaoming L., Yujin G., Xingwang Z. (2020). Sperm DNAfragmentation index with unexplained recurrent spontaneous abortion: Asystematic review and meta-analysis. J. Gynecol. Obstet. Hum. Reprod..

[35] Miyakis S., Lockshin M.D., Atsumi T., Branch D.W., Brey R.L., Cervera R., Derksen R.H.W.M., De Groot P.G., Koike T., Meroni P.L. (2006). International consensus statement on an update of the classification criteria for definite antiphospholipid syndrome (APS). J. Thromb. Haemost..

[36] Meroni P.L., Borghi M.O., Raschi E., Tedesco F. (2011). Pathogenesis of antiphospholipid syndrome: Understanding the antibodies. Nat. Rev. Rheumatol..

[37] Di Simone N., Castellani R., Caliandro D., Caruso A. (2001). Monoclonal Anti-Annexin V Antibody Inhibits Trophoblast Gonadotropin Secretion and Induces Syncytiotrophoblast Apoptosis. Biol. Reprod..

[38] Kaneko K., Ozawa N., Murashima A. (2022). Obstetric anti-phospholipid syndrome: From pathogenesis to treatment. Immunol. Med..

[39] Stevens S.M., Woller S.C., Bauer K.A., Kasthuri R., Cushman M., Streiff M., Lim W., Douketis J.D. (2016). Guidance for the evaluation and treatment of hereditary and acquired thrombophilia. J. Thromb. Thrombolysis.

[40] Simcox L.E., Ormesher L., Tower C., Greer I.A. (2015). Thrombophilia and Pregnancy Complications. Int. J. Mol. Sci..

[41] Torabi R., Zarei S., Zeraati H., Zarnani A.H., Akhondi M.M., Hadavi R., Shiraz E.S., Jeddi-Tehrani M. (2012). Combination of Thrombophilic Gene Polymorphisms as a Cause of Increased the Risk of Recurrent Pregnancy Loss. J. Reprod. Infertil..

[42] Deng Y.-J., Liu S.-J., Zhao M., Zhao F., Guo J., Huang Y.-X. (2022). Research trends and hotspots of recurrent pregnancy loss with thrombophilia: A bibliometric analysis. BMC Pregnancy Childbirth.

[43] Rey E., Kahn S.R., David M., Shrier I. (2003). Thrombophilic disorders and fetal loss: A meta-analysis. Lancet.

[44] Silver R.M., Zhao Y., Spong C.Y., Sibai B., Wendel G.J., Wenstrom K., Samuels P., Caritis S.N., Sorokin Y., Miodovnik M. (2010). Prothrombin Gene G20210A Mutation and Obstetric Complications. Obstet. Gynecol..

[45] Lissalde-Lavigne G., Fabbro-Peray P., Cochery-Nouvellon E., Mercier E., Ripart-Neveu S., Balducchi J.-P., Daures J.-P., Perneger T., Quere I., Dauzat M. (2005). Factor V Leiden and prothrombin G20210A polymorphisms as risk factors for miscarriage during a first intended pregnancy: The matched case-control “NOHA first” study. J. Thromb. Haemost..

[46] Dizon-Townson D., Miller C., Sibai B., Spong C.Y., Thom E., Wendel G., Wenstrom K., Samuels P., Cotroneo M.A., Moawad A. (2005). The Relationship of the Factor V Leiden Mutation and Pregnancy Outcomes for Mother and Fetus. Obstet. Gynecol..

[47] De Jong P.G., Goddijn M., Middwldorp S. (2011). Testing for inherited thrombophilia in recurrent miscarriage. Sem. Reprod. Med..

[48] Moffett A., Regan L., Braude P. (2004). Natural killer cells, miscarriage, and infertility. BMJ.

[49] Le Bouteiller P., Piccinni M.P. (2008). Human NK cells in pregnant uterus: Why there?. Am. J. Reprod. Immunol..

[50] Tuckerman E., Laird S.M., Prakash A., Li T.C. (2007). Prognostic value of the measurement of uterine natural killer cells in theendometrium of women with recurrent miscarriage. Hum. Reprod..

[51] Kuon R.J., Müller F., Vomstein K., Weber M., Hudalla H., Rösner S., Strowitzki T., Markert U., Daniel V., Toth B. (2017). Pre-Pregnancy Levels of Peripheral Natural Killer Cells as Markers for Immunomodulatory Treatment in Patients with Recurrent Miscarriage. Arch. Immunol. Ther. Exp..

[52] Comins-Boo A., Cristóbal I., Fernández-Arquero M., de Frías E.R., Urrutia M.C., Suárez L.P., Escorial P.G., Herráiz M., Sánchez-Ramón S. (2021). Functional NK surrogate biomarkers for inflammatory recurrent pregnancy loss and recurrent implantation failure. Am. J. Reprod. Immunol..

[53] Vomstein K., Feil K., Strobel L., Aulitzky A., Hofer-Tollinger S., Kuon R.-J., Toth B. (2021). Immunological Risk Factors in Recurrent Pregnancy Loss: Guidelines Versus Current State of the Art. J. Clin. Med..

[54] Ying Y., Zhong Y.-P., Zhou C.-Q., Xu Y.-W., Ding C.-H., Wang Q., Li J., Shen X.-T. (2013). A Further Exploration of the Impact of Antinuclear Antibodies on *In Vitro* Fertilization-Embryo Transfer Outcome. Am. J. Reprod. Immunol..

[55] D’Ippolito S., Ticconi C., Tersigni C., Garofalo S., Martino C., Lanzone A., Scambia G., Di Simone N. (2020). The pathogenic role of autoantibodies in recurrent pregnancy loss. Am. J. Reprod. Immunol..

[56] Veglia M., D’Ippolito S., Marana R., Di Nicuolo F., Castellani R., Bruno V., Fiorelli A., Ria F., Maulucci G., De Spirito M. (2015). Human IgG Antinuclear Antibodies Induce Pregnancy Loss in Mice by Increasing Immune Complex Deposition in Placental Tissue: *In Vivo* Study. Am. J. Reprod. Immunol..

[57] Zeng M., Wen P., Duan J. (2019). Association of antinuclear antibody with clinical outcome of patients undergoing in vitro fertilization/intracytoplasmic sperm injection treatment: A meta-analysis. Am. J. Reprod. Immunol..

[58] Nørgaard-Pedersen C., Steffensen R., Kesmodel U.S., Christiansen O.B. (2023). A combination of the HLA-DRB1*03 phenotype and low plasma mannose-binding lectin predisposes to autoantibody formation in women with recurrent pregnancy loss. Front. Immunol..

[59] Jørgensen M.M., Bæk R., Sloth J., Varming K., Christiansen O.B., Ditlevsen N.E., Rajaratnam N. (2020). Treatment with intravenous immunoglobulin increases the level of small EVs in plasma of pregnant women with recurrent pregnancy loss. J. Reprod. Immunol..

[60] Rajaratnam N., Ditlevsen N.E., Sloth J.K., Bæk R., Jørgensen M.M., Christiansen O.B. (2021). Extracellular Vesicles: An Important Biomarker in Recurrent Pregnancy Loss?. J. Clin. Med..

[61] Yang L., Cambou M.C., Segura E.R., De Melo M.G., Santos B.R., Varella I.R.D.S., Nielsen-Saines K. (2022). Patterns of pregnancy loss among women living with and without HIV in Brazil, 2008–2018. AJOG Glob. Rep..

[62] Cates J.E., Westreich D., Edmonds A., Wright R.L., Minkoff H., Colie C., Greenblatt R.M., Cejtin H.E., Karim R., Haddad L.B. (2015). The Effects of Viral Load Burden on Pregnancy Loss among HIV-Infected Women in the United States. Infect. Dis. Obstet. Gynecol..

[63] Cerovac A., Brigic A., Softic D., Barakovic A., Adzajlic S. (2020). Uncontrolled Acute Intermittent Porphyria as a Cause of Spontaneous Abortion. Med Arch..

[64] Pandey U., Dixit V.K. (2013). Acute intermittent porphyria in pregnancy: A case report and review of literature. J. Indian Med Assoc..

[65] Nørgaard-Pedersen C., Rom L.H., Steffensen R., Kesmodel U.S., Christiansen O.B. (2022). Plasma level of mannose-binding lectin is associated with the risk of recurrent pregnancy loss but not pregnancy outcome after the diagnosis. Hum. Reprod. Open.

[66] Lindbohm M.-L., Sallmén M., Taskinen H. (2002). Effects of exposure to environmental tobacco smoke on reproductive health. Scand. J. Work. Environ. Health.

[67] Boots C., Stephenson M. (2011). Does Obesity Increase the Risk of Miscarriage in Spontaneous Conception: A Systematic Review. Semin. Reprod. Med..

[68] Kesmodel U., Wisborg K., Olsen S.F., Henriksen T.B., Secher N.J. (2002). Moderate alcohol intake in pregnancy and the risk of spontaneous abortion. Alcohol Alcohol..

[69] Robberecht C., Schuddinck V., Fryns J.-P., Vermeesch J.R. (2009). Diagnosis of miscarriages by molecular karyotyping: Benefits and pitfalls. Genet. Med..

[70] Kudesia R., Li M., Smith J., Patel A., Williams Z. (2014). Rescue karyotyping: A case series of array-based comparative genomic hybridization evaluation of archival conceptual tissue. Reprod. Biol. Endocrinol..

[71] Mathur N., Triplett L., Stephenson M.D. (2014). Miscarriage chromosome testing: Utility of comparative genomic hybridization with reflex microsatellite analysis in preserved miscarriage tissue. Fertil. Steril..

[72] Shamseldin H.E., Swaid A., Alkuraya F.S. (2013). Lifting the lid on unborn lethal Mendelian phenotypes through exome sequencing. Genet. Med..

[73] Jaslow C.R., Kutteh W.H. (2013). Effect of prior birth and miscarriage frequency on the prevalence of acquired and congenital uterine anomalies in women with recurrent miscarriage: A cross-sectional study. Fertil. Steril..

[74] Grimbizis G.F., Sardo A.D.S., Saravelos S.H., Gordts S., Exacoustos C., Van Schoubroeck D., Bermejo C., Amso N.N., Nargund G., Timmermann D. (2016). The Thessaloniki ESHRE/ESGE consensus on diagnosis of female genital anomalies. Gynecol. Surg..

[75] Saravelos S.H., Cocksedge K.A., Li T.-C. (2008). Prevalence and diagnosis of congenital uterine anomalies in women with reproductive failure: A critical appraisal. Hum. Reprod. Updat..

[76] Ludwin A., Ludwin I., Banas T., Knafel A., Miedzyblocki M., Basta A. (2011). Diagnostic accuracy of sonohysterography, hysterosalpingography and diagnostic hysteroscopy in diagnosis of arcuate, septate and bicornuate uterus. J. Obstet. Gynaecol. Res..

[77] Caliskan E., Ozkan S., Cakiroglu Y., Sarisoy H.T., Corakci A., Ozeren S. (2010). Diagnostic accuracy of real-time 3D sonography in the diagnosis of congenital Mullerian anomalies in high-risk patients with respect to the phase of the menstrual cycle. J. Clin. Ultrasound.

[78] Chan Y., Jayaprakasan K., Zamora J., Thornton J., Raine-Fenning N., Coomarasamy A. (2011). The prevalence of congenital uterine anomalies in unselected and high-risk populations: A systematic review. Hum. Reprod. Updat..

[79] Triggianese P., Perricone C., Perricone R., De Carolis C. (2015). Prolactin and Natural Killer Cells: Evaluating the Neuroendocrine-immune Axis in Women with Primary Infertility and Recurrent Spontaneous Abortion. Am. J. Reprod. Immunol..

[80] Li W., Ma N., Laird S.M., Ledger W.L., Li T.C. (2013). The relationship between serum prolactin concentration and pregnancy outcome in women with unexplained recurrent miscarriage. J. Obstet. Gynaecol..

[81] Bates S.M., Greer I.A., Middeldorp S., Veenstra D.L., Prabulos A.-M., Vandvik P.O. (2012). VTE, Thrombophilia, Antithrombotic Therapy, and Pregnancy: Antithrombotic Therapy and Prevention of Thrombosis, 9th ed: American College of Chest Physicians Evidence-Based Clinical Practice Guidelines. Chest.

[82] Kristoffersen A.H., Petersen P.H., Røraas T., Sandberg S. (2017). Estimates of Within-Subject Biological Variation of Protein C, Antithrombin, Protein S Free, Protein S Activity, and Activated Protein C Resistance in Pregnant Women. Clin. Chem..

[83] Huynh K., Kahwaji C.I. (2023). HIV Testing.

[84] Drakeley A.J., Roberts D., Alfirevic Z. (2003). Cervical stitch (cerclage) for preventing pregnancy loss in women. Cochrane Database Syst. Rev..

[85] Hirahara F., Andoh N., Sawai K., Hirabuki T., Uemura T., Minaguchi H. (1998). Hyperprolactinemic recurrent miscarriage and results of randomized bromocriptine treatment trials. Fertil. Steril..

[86] Andrade C. (2016). Major Malformation Risk, Pregnancy Outcomes, and Neurodevelopmental Outcomes Associated With Metformin Use During Pregnancy. J. Clin. Psychiatry.

[87] Showell M.G., Mackenzie-Proctor R., Brown J., Yazdani A., Stankiewicz M.T., Hart R.J. (2014). Antioxidants for male subfertility. Cochrane Database Syst. Rev..

[88] Empson M.B., Lassere M., Craig J.C., Scott J.R. (2005). Prevention of recurrent miscarriage for women with antiphospholipid antibody or lupus anticoagulant. Cochrane Database Syst. Rev..

[89] Scarpellini F., Klinger F.G., Rossi G., Sbracia M. (2019). Immunohistochemical Study on the Expression of G-CSF, G-CSFR, VEGF, VEGFR-1, Foxp3 in First Trimester Trophoblast of Recurrent Pregnancy Loss in Pregnancies Treated with G-CSF and Controls. Int. J. Mol. Sci..

[90] Porter T.F., LaCoursiere Y., Scott J.R. (2006). Immunotherapy for recurrent miscarriage. Cochrane Database Syst. Rev..

[91] Christiansen O.B., Kolte A.M., Krog M.C., Nielsen H.S., Egerup P. (2019). Treatment with intravenous immunoglobulin in patients with recurrent pregnancy loss: An update. J. Reprod. Immunol..

[92] Yamada H., Deguchi M., Saito S., Takeshita T., Mitsui M., Saito T., Nagamatsu T., Takakuwa K., Nakatsuka M., Yoneda S. (2022). Intravenous immunoglobulin treatment in women with four or more recurrent pregnancy losses: A double-blind, randomised, placebo-controlled trial. Eclinicalmedicine.

[93] Stephenson M.D., Kutteh W.H., Purkiss S., Librach C., Schultz P., Houlihan E., Liao C. (2010). Intravenous immunoglobulin and idiopathic secondary recurrent miscarriage: A multicentered randomized placebo-controlled trial. Hum. Reprod..

[94] Bailey H., Zash R., Rasi V., Thorne C. (2018). HIV treatment in pregnancy. Lancet HIV.

[95] WHO (2016). Consolidated Guidelines on the Use of Antiretroviral Drugs for Treating and Preventing HIV Infection: Recommendations for a Public Health Approach.

[96] Watts T., Stockman L., Martin J., Guilfoyle S.M., Vergeront J.M., Zahner S. (2020). Estimates of Prenatal HIV, Hepatitis B Virus, and Hepatitis C Virus Testing Among Pregnant People Enrolled in Wisconsin Medicaid, 2011–2015. Matern. Child Health J..

[97] De Jong P.G., Quenby S., Bloemenkamp K.W., Braams-Lisman B.A., de Bruin J.P., Coomarasamy A., David M., DeSancho M.T., van der Heijden O.W., Hoek A. (2015). ALIFE2 study: Low-molecular-weight heparin for women with recurrent miscarriage and inherited thrombophilia—Study protocol for a randomized controlled trial. Trials.

[98] Hisano M., Nakagawa K., Kwak-Kim J., Sugiyama R., Sago H., Yamaguchi K. (2022). Changes in the T-helper 1 and 2 cell populations during pregnancy in tacrolimus-treated women with repeated implantation failure and recurrent pregnancy loss. Hum. Fertil..

[99] Nakagawa K., Kuroda K., Sugiyama R., Yamaguchi K. (2017). After 12 consecutive miscarriages, a patient received immunosuppressive treatment and delivered an intact baby. Reprod. Med. Biol..

[100] Casella G., Orfanotti G., Giacomantonio L., Di Bella C., Crisafulli V., Villanacci V., Baldini V., Bassotti G. (2016). Celiac disease and obstetrical-gynecological contribution. Gastroenterol. Hepatol. Bed Bench.

[101] Sharshiner R., Romero S.T., Bardsley T.R., Branch D.W., Silver R.M. (2013). Celiac disease serum markers and recurrent pregnancy loss. J. Reprod. Immunol..

[102] Kutteh M.A., Abiad M., Norman G.L., Kutteh W.H. (2019). Comparison of celiac disease markers in women with early recurrent pregnancy loss and normal controls. Am. J. Reprod. Immunol..

[103] Masucci L., D’ippolito S., De Maio F., Quaranta G., Mazzarella R., Bianco D.M., Castellani R., Inversetti A., Sanguinetti M., Gasbarrini A. (2023). Celiac Disease Predisposition and Genital Tract Microbiota in Women Affected by Recurrent Pregnancy Loss. Nutrients.

[104] ClinicalTrials.gov Identifier: NCT03305263. NCT03305263.

[105] ClinicalTrials.gov Identifier: NCT05510622. NCT05510622.

